# Foundations of data-driven medicinal chemistry

**DOI:** 10.4155/fsoa-2018-0057

**Published:** 2018-06-28

**Authors:** Jürgen Bajorath

**Affiliations:** 1 Department of Life Science Informatics, B-IT, LIMES Program Unit Chemical Biology & Medicinal Chemistry, Rheinische Friedrich-Wilhelms-Universität, Endenicher Allee 19c, D-53115 Bonn, Germany

**Keywords:** activity data, analytics, big data, bioactive compounds, data integration, data-driven medicinal chemistry, data mining, predictive models

Progress in hit-to-lead and lead optimization (LO) projects largely depends on intuition, experience, and the individual contributions of practicing medicinal chemists. LO involves the sequential generation of analogs of a given lead (or of multiple leads in parallel) to further improve compound potency and other relevant molecular properties. It is a resource-intense and time-consuming process, which is often perceived to be more of an art form than rigorous science. Decisions around which compounds to make next may or may not be supported by quantitative structure–activity relationship analysis or other computational design approaches. It is rare, however, that compound activity data available for the same or closely related targets are taken into consideration, even if such data were previously generated in-house and not externally. This situation should be critically viewed at times when increasingly large amounts of new compounds and activity data become publicly available and ‘big data’ begin to emerge in biological and medicinal chemistry [1–5]. Regardless of whether one is in favor of incorporating informatics approaches into medicinal chemistry programs or not, neglecting opportunities for data-driven chemistry is a serious issue and hinders the further development of medicinal chemistry as a scientific discipline.

## Data-driven medicinal chemistry

Data-driven medicinal chemistry (DDMC) currently is more or less in its conceptual stages and far from being a routine approach. DDMC can be rationalized as the application of computational (informatics) methods for data integration, representation, analysis and knowledge extraction to enable decision making in medicinal chemistry on the basis of internal and public domain data. Such data primarily include – but are not limited to – compound structures, activity and pharmacological data from which structure–activity relationships (SARs) can be extracted and compared to guide hit-to-lead and LO efforts.

DDMC is desirable because it is less subjective and based upon a larger knowledge base than conventional LO efforts. However, comprehensive data analysis usually suggests alternative solutions, which challenge the common practice of medicinal chemistry. This may explain why investigators are often reluctant to accept DDMC concepts. Moreover, given established reward structures in the pharmaceutical industry, practicing chemists might fear that data-driven decisions devalue individual contributions. Such views complement scientific and technical challenges to devise and implement DDMC infrastructures and protocols.

## Historical data

Learning from data accumulating in-house over time still is an exception rather than the rule in the pharmaceutical industry. This results in the presence of largely unexplored sources of drug discovery knowledge. Exploring historical data requires resources, which are often not dedicated in pharmaceutical environments where progress is rewarded, not retrospective analysis. This translates into missed opportunities. Furthermore, if only a fraction of historical data accumulated across the industry would be made available to academia and data science institutions, for example, as a part of open science and innovation initiatives [6,7], it would most likely catalyze a widespread development of data-driven discovery strategies. The potential IP relevance of accumulated in-house data, which is a prime argument against data sharing by pharmaceutical companies, is often overestimated, especially if the data originated from therapeutic areas that are no longer investigated internally. Learning from such data may be more profitable than little-supported IP expectations. Maintaining an aura of data secrecy works against a culture of proactive and comparative data analysis and also prevents the consideration of external data that are not IP relevant and are therefore thought to be ‘less valuable’.

## Data integration

For data-driven medicinal chemistry, integration of internal and external data is a must [3]. Major public repositories for compounds and activity data from the medicinal chemistry literature and screening campaigns have been established and can be utilized [8,9]. However, internal informatics infrastructures for data extraction and integration continue to be unavailable in many pharmaceutical environments. In addition, there are other factors that hinder data integration. For example, data quality is a major concern in pharmaceutical research and development [3] and the quality of external data is often questioned, making medicinal chemists and others reluctant to seriously consider such data. Such concerns are principally valid but must be overcome for data integration, for example, by implementing internal curation protocols. Furthermore, data from public sources are typically heterogeneous and must be made available in a form that is useful to practitioners. Consistent data representation including visualization is a challenging task. For this and other requirements associated with data integration, representation and knowledge extraction, the data science field [10,11] provides a highly relevant reference point that merits close consideration.

## Data science

Computational groups are challenged to provide external and internal data in an easily accessible form. However, the problem is more general. In data science, community-wide standards and tools for data processing and knowledge extraction are available [10] but in chemistry, such standards and infrastructures are lacking. Accordingly, they should be adopted by data science experts, not chemists. Moreover, in data science, the context dependence of data analysis and knowledge extraction is as much appreciated as the need to generalize conclusions drawn from data analysis [10]. Generalization requires abstraction from individual datasets and the consideration of context-dependent factors that influence data and knowledge extracted from them. These issues must be carefully evaluated when devising strategies for data-driven medicinal chemistry, calling again for an immediate involvement of data scientists.

In medicinal chemistry, the context-dependence of data analysis also applies, for example, given individual compound classes, targets, their biology or specific requirements for therapeutic intervention. However, compared with other fields, the need for data generalization is reduced. For example, it is typically not required to transfer strategies successfully applied in a particular LO campaign to another with different requirements. Rather, the key task is enabling data-driven decisions during LO, regardless of the specifics of the project.

## Data mining & modeling

It is often assumed that knowledge extraction from data inevitably involves machine learning and the derivation of predictive models [3]. This also causes reluctance by practitioners who do not wish to depend on computational predictions they do not understand. However, learning from data does not necessarily imply relying on black box predictions using complicated computational methods [12]. In data science, models for data rationalization and prediction are distinguished [11]. Differently put, data analytics should be differentiated from predictive modeling. For example, in SARs analysis, the inclusion of external data typically helps to better understand SARs and molecular regions that determine them. Whether or not new compounds are predicted in this context becomes an issue beyond data analytics.

## Going forward

It is the author's opinion that the design and implementation of data-driven strategies will be critical for the future of medicinal chemistry as a scientific discipline. The key questions on the agenda for medicinal chemists have not changed for many years. For example, such questions include when sufficient compounds have been made in an LO project and no further progress can be expected or if an initially observed SAR can be further evolved. Only DDMC will be able to successfully address such questions, which cannot be answered from first principles. To establish DDMC, data scientists will need to be added to pharmaceutical environments. The presence of a few computational chemists dedicated to DDMC issues will not be enough. Moreover, it will be required to increasingly incorporate informatics education in – traditionally conservative – chemistry curricula to prepare future generations of chemists for the challenges and opportunities of DDMC.
